# Idiopathic inferior mesenteric arteriovenous fistula with bleeding diarrhea: A case report

**DOI:** 10.1097/MD.0000000000046214

**Published:** 2026-05-12

**Authors:** Congcong Shi, Xuan Zheng, Lihong Chen, Shenfeng Wu, Ziyou Zhong, Yufeng Xing

**Affiliations:** a The Fourth Clinical Medicine School of Guangzhou University of Chinese Medicine, Shenzhen, Guangdong, China; b The First Clinical Medicine School of Guangzhou University of Chinese Medicine, Guangzhou, Guangdong, China; c Department of Surgery, Shenzhen Traditional Chinese Medicine Hospital, Shenzhen, Guangdong, China; d Department of Gastroenterology, Shenzhen Traditional Chinese Medicine Hospital, Shenzhen, Guangdong, China; e Department of Hepatology, Shenzhen Traditional Chinese Medicine Hospital, Shenzhen, Guangdong, China.

**Keywords:** arteriovenous fistula, bowel ischemia, case report, inferior mesenteric arteriovenous fistula, open laparotomy low anterior resection, watery and bleeding diarrhea

## Abstract

**Rationale:**

The inferior mesenteric arteriovenous fistula (IMAV AVF) is an anomalous connection between the arteries and veins of the mesentery, bypassing the capillary network and typically involving the hepatic, superior mesenteric, and splenic arteries.

**Patient concerns:**

A 64-year-old previously healthy male presents with a 2-month history of bloody and watery diarrhea with lower abdominal pain, and a notable 10-kg weight loss.

**Diagnoses:**

A diagnosis of ischemic colitis (IC) was supported by a colonoscopy that revealed diffuse ulcerations and stenosis from the sigmoid-descending colon junction to the upper rectum. The nidus of the inferior mesenteric artery (IMA)V AVF was observed by digital subtraction angiography to emerge from the IMA and empty into the inferior mesenteric vein. IC and IMAV AVF were confirmed pathologically.

**Interventions:**

We performed a low anterior resection to completely resect the AVM and postoperative pathology confirmed IMAV AVF. An end colostomy was created.

**Outcomes:**

The patient was ultimately diagnosed with IMAV AVF.

**Lessons:**

The diagnosis of exclusion is commonly used for idiopathic IMAV AVF. Clinicians should consider patients exhibiting gastrointestinal symptoms similar to those associated with IC as potential candidates for the diagnosis of this disease. Based on the patient’s condition, rational selection of endovascular and surgical treatment is necessary.

## 1. Introduction

The inferior mesenteric arteriovenous fistula (IMAV AVF) is an anomalous connection between the arteries and veins of the mesentery that bypassing the capillary network and typically involving the hepatic, superior mesenteric, and splenic arteries. To date, approximately 40 cases of IMAV AVF have been documented in the literature.^[1]^ Although it is a rare condition, IMAV AVF is a significant cause of lower gastrointestinal (GI) bleeding. Clinical manifestations may arise from ischemic, congestive, or hemodynamic mechanisms, leading to symptoms such as abdominal pain, abdominal masses, GI bleeding, colonic ischemia, and portal hypertension.^[2]^ Patients often present with melena and hemorrhoids.

Arteriovenous fistulas (AVFs) are abnormal arteriovenous connections that bypass the capillary bed^[3]^ and can be congenital, idiopathic, or acquired. Congenital IMAV AVF may result from developmental arrest or circulatory anomalies during embryonic development, leading to vascular malformations. The rupture of a congenital arterial aneurysm near a vein can also lead to idiopathic AVFs.^[4]^ Acquired IMAV AVF is often secondary to trauma, surgical complications, intra-abdominal arteries rupture, infections, or age-related vascular degeneration.^[5–7]^ Given the diverse etiologies and potential complications of IMAV AVF, accurate diagnosis is critical for guiding targeted treatment.

Color Doppler flow imaging is the preferred screening modality for IMAV AVF, as it enables objective assessment of flow velocity, rate, and other hemodynamic indexes. Digital subtraction angiography remains the gold standard for definitive diagnosis, typically demonstrating nodular vascular malformations and dilated draining veins. Treatment of IMAV AVF focuses on alleviating venous hypertension and is primarily achieved through interventional radiology (IVR), endoscopy, or surgery.

Herein, we report a case of idiopathic IMAV AVF presenting with watery, bleeding diarrhea, detailing the diagnostic and therapeutic journey to provide clinicians with a practical reference.

## 2. Case presentation

### 2.1. History

A 64-year-old male presented with a 2-month history of diarrhea for further evaluation. The patient’s primary complaints included lower abdominal pain, watery and bloody diarrhea, and a 10-kg weight loss. Before this episode, the patient did not have any underlying health conditions and denied any previous similar symptoms, history of abdominal surgery, trauma, or clinical evidence of portal hypertension. Family and genetic histories were non.

### 2.2. Examination findings

Upon physical examination, auscultation and abdominal visualization showed no evident abnormalities. Palpation, however, revealed a firm, immovable mass that measured about 20 by 9 cm, with tenderness in the lower left quadrant.

When he was admitted, his hemoglobin concentration was 115g/L, white blood cell count was 21.94*109/L, neutrophil granulocyte count was 20.20*109/L, high-sensitivity c-reactive protein was 147.05 mg/L, and prothrombin time was 15.9 seconds. Additionally, erythrocyte, hemoglobin, and transferrin tests on stool samples were positive.

**Figure 1.**
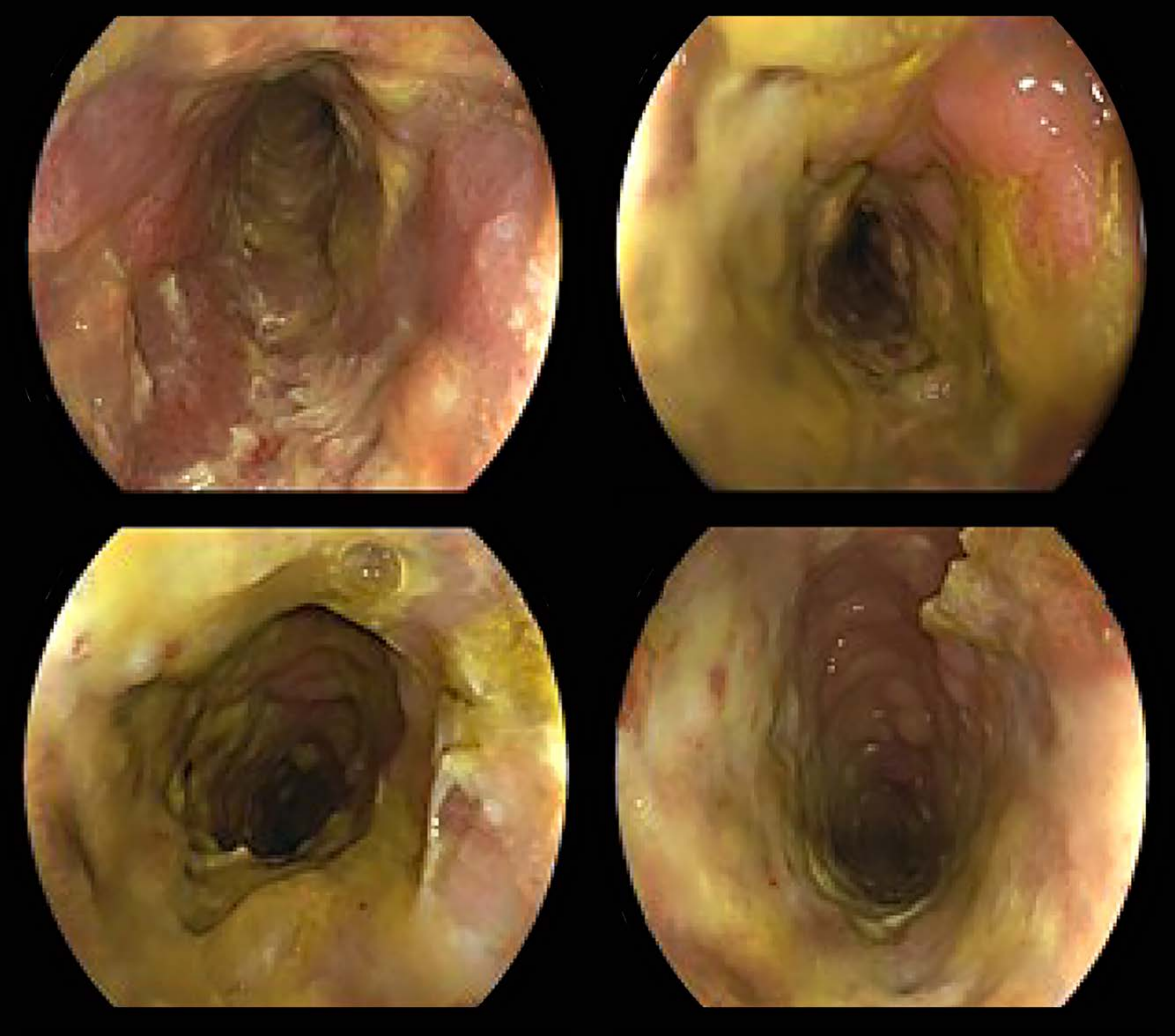
The segment from the sigmoid-descending colon junction to the upper rectum was characterized by ischemic enteritis with concomitant stenosis.

**Figure 2.**
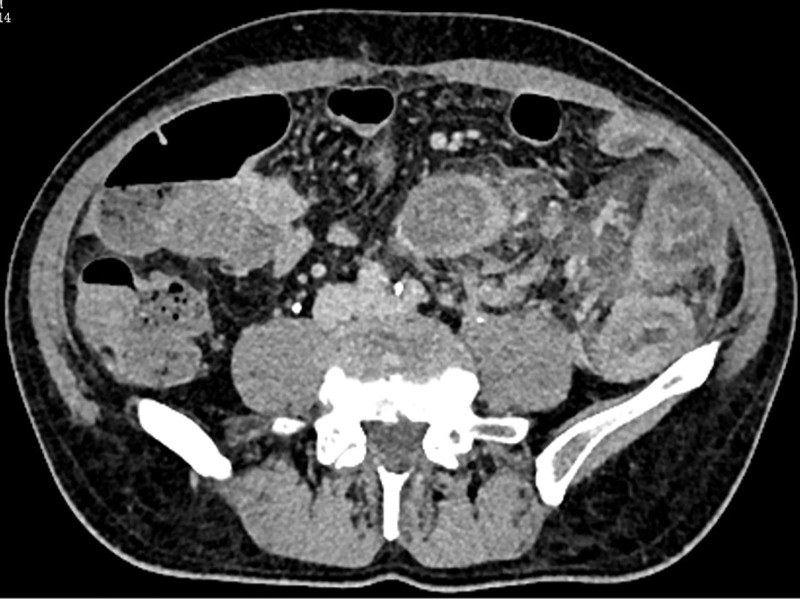
Marked circumferential thickening of the intestinal wall is appreciated in the region supplied by the IMA (arrow). IMA = inferior mesenteric artery.

**Figure 3.**
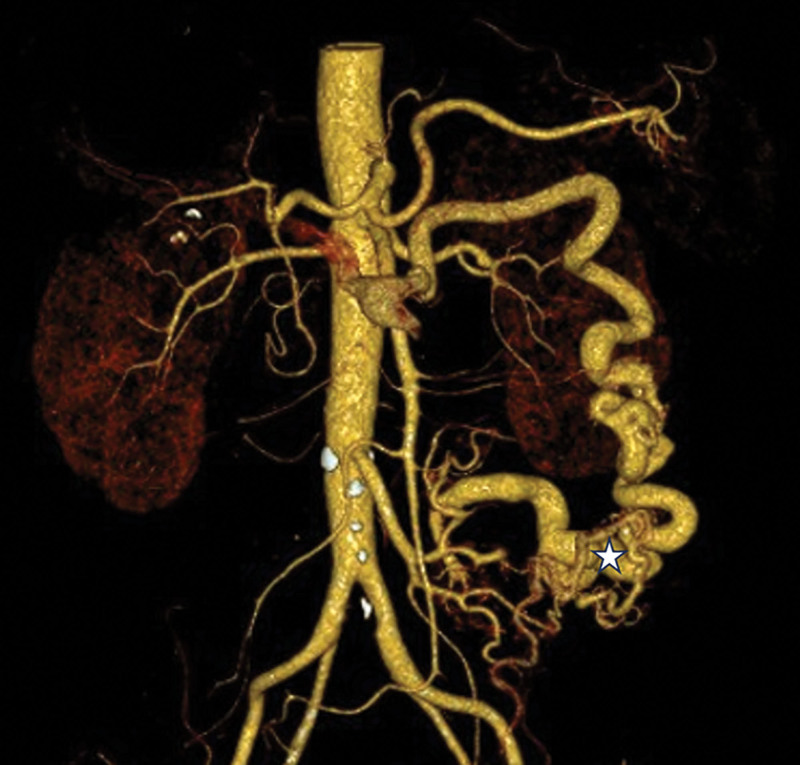
Tortuous dilated arteriovenous malformation (star) were noted in the vascular arcade supplied by the inferior mesenteric artery, spanning the region from the descending colon-sigmoid junction to the upper rectal segment.

**Figure 4.**
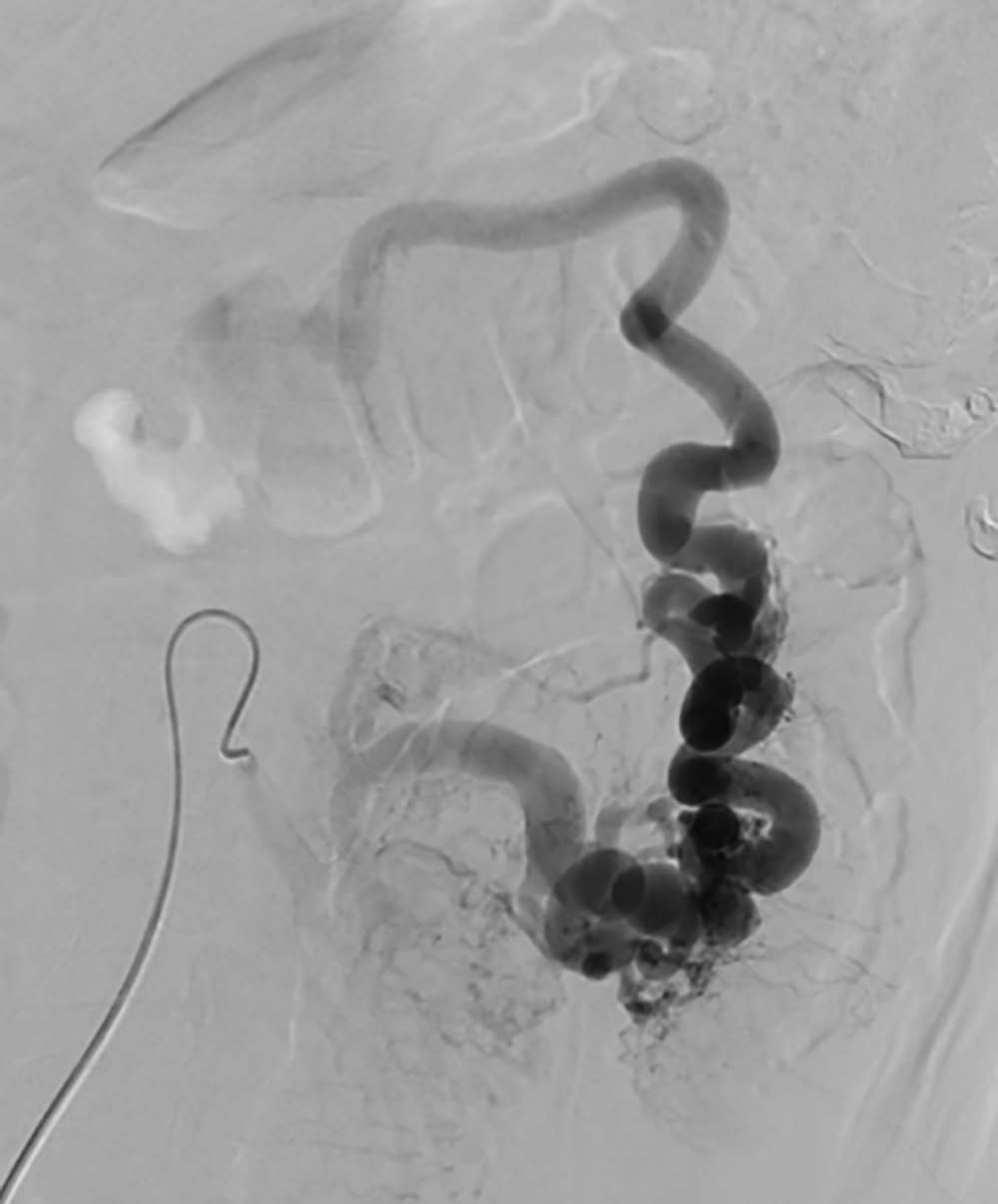
Arteriography reveals markedly dilated vessels with high-velocity flow.

Electrolytes, pancreatic enzymes, cardiac enzymes, and hepatic or kidney function were all free of notable anomalies. A diagnosis of ischemic colitis (IC) was supported by a colonoscopy that showed diffuse ulcerations and stenosis from the sigmoid-descending colon junction to the upper rectum (Fig. 1). Signs of ischemia, including thickening of the intestinal wall and dilatation of blood vessels supplied by the inferior mesenteric artery (IMA), were verified by a contrast-enhanced computed tomography (CT) scan (Fig. 2). For definitive diagnosis, multiphasic CT (MDCT) angiography of the abdominal aorta, superior mesenteric vein, and IMA was performed (Fig. 3). Angiography identified a nidus of the IMAV AVF arising from the IMA and draining into the inferior mesenteric vein (Fig. 4). No significant abnormalities were noted in the pancreas, liver or portal vein.

### 2.3. Treatment course

**Figure 5.**
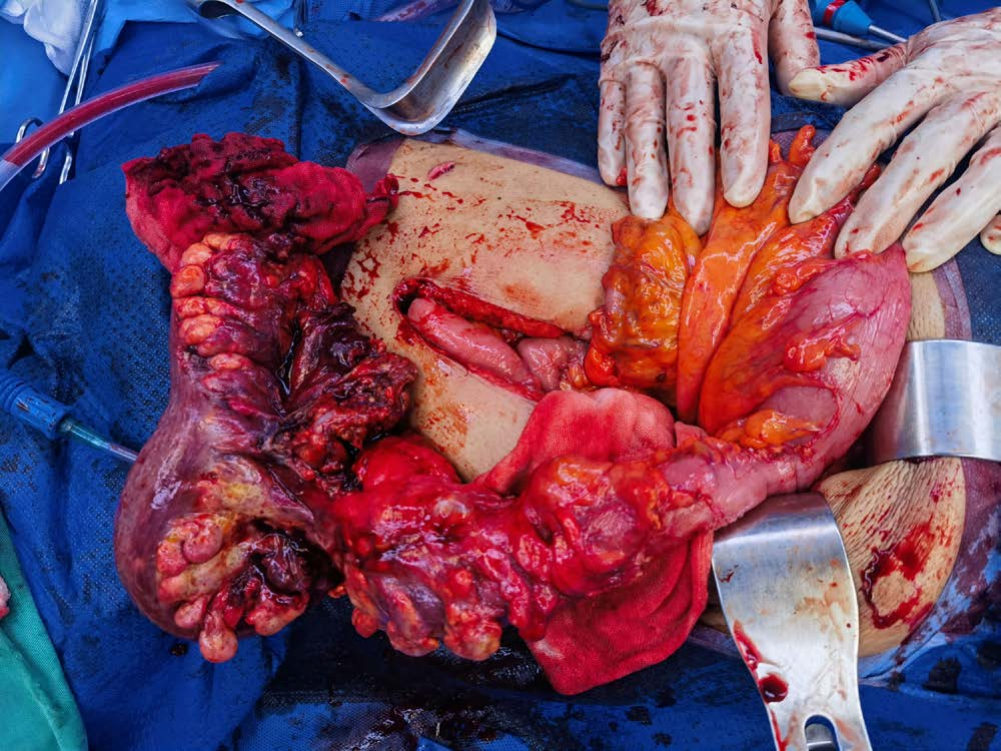
The descending sigmoid colon involved by tortuous vessels was resected.

Given the large fistula size (>8 mm), high flow rate, multiple feeding vessels, and risk of colonic ischemia, surgical resection was chosen over angiographic embolization. Initially, a laparoscopic low anterior resection was attempted to completely resect the arteriovenous malformation (AVM). But laparoscopic observation revealed extensive edema, thickening, stenosis, and rigidity from the rectum to the splenic flexure of the descending colon and hyperplasia of blood vessels around the lesion. Exploratory laparoscopy was converted to an open operation due to the extensively inflamed and thickened left colonic mesentery. A 15-cm midline incision was made, and the descending, sigmoid colon was resected (Fig. 5). Additionally, since the CT scan revealed gallstones that were confirmed during the operation but were not associated with the patient’s gastrointestinal symptoms, and there was no evidence of cholecystitis or biliary obstruction. Following informed consent from the patient and their family, we proceeded with cholecystectomy. IC and AVF were confirmed in pathology. An end colostomy was created to protect the anastomosis.

### 2.4. Follow-up

**Figure 6.**
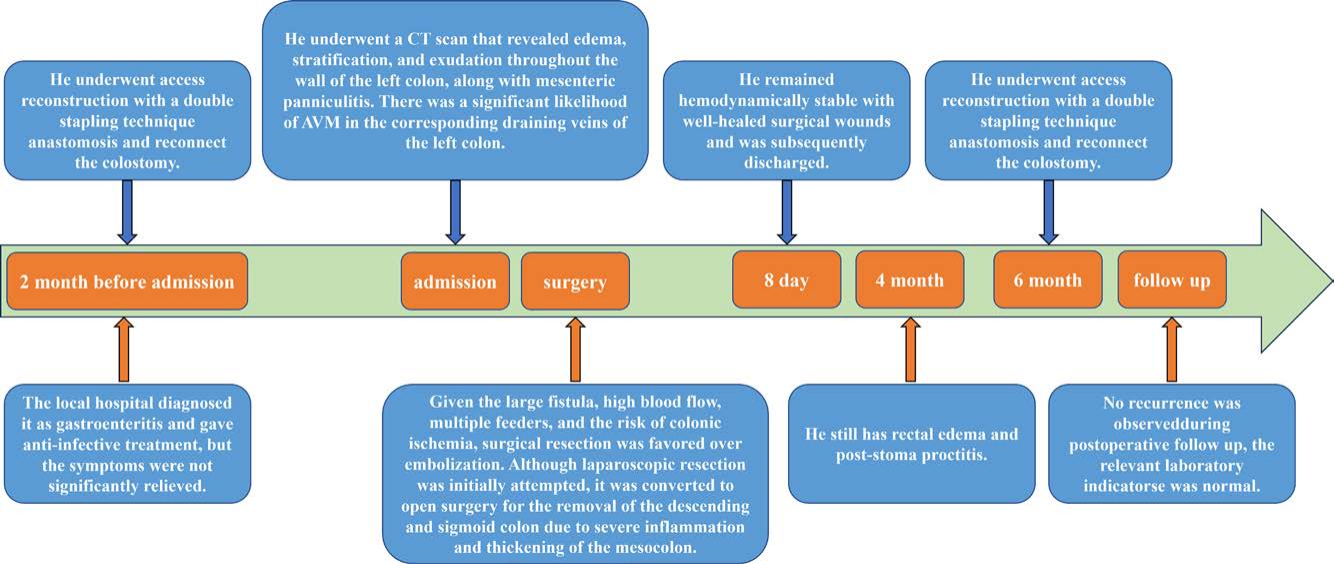
The timeline of this case.

The patient achieved an uneventful postoperative recovery and was discharged on postoperative day 8. Stoma reversal was scheduled for 4 months postoperatively; however, severe rectal edema and poststomy proctitis at the planned time led to a suboptimal surgical outcome, prompting colostomy reinstatement at 6 months postoperatively. Serial colonoscopies and abdominal CT scans performed during this interval yielded no remarkable findings. At 6 months following the initial operation, the patient underwent access reconstruction with a double stapling technique anastomosis and reconnect the colostomy. The patient was discharged in good general condition on postoperative day 22. We used a timeline to visually display theentire treatment process (Fig. 6).

## 3. Discussion

Our patient was previously in good health with no underlying illnesses and no history of abdominal surgery or trauma. Accordingly, an idiopathic IMAV AVF was hypothesized as the etiological factor.

IMAV AVF is typically associated with diminished arterial blood supply to the tissues surrounding the fistula and increased venous blood flow distal to the fistula, resulting in various clinical symptoms, including abdominal pain, lower gastrointestinal bleeding, portal hypertension, and IC.^[4,8]^ Our patient presented with typical symptoms of abdominal pain in the left lower abdomen along with a palpable and visible mass. Additionally, the patient experienced frequent diarrhea, primarily with muco-bloody stools. The abdominal pain is attributed to arterial perfusion shunting in the left and venous circulation congestion, and the congested colon may form a palpable abdominal mass. Intestinal mucosal vascular congestion and IC were identified as potential drivers of the patient’s lower GI bleeding. Additionally, arteriovenous shunting from the IMAVF to the portal system induced portal hypertension by augmenting portal inflow, eliciting a compensatory increase in hepatic vascular resistance. IMAV AVF induces IC by reducing arterial blood flow to the mucosa due to a steal phenomenon and causing congestive submucosal edema from venous hypertension.^[4,9,10]^

As a prevalent acute surgical entity, IC typically presents with abdominal pain and lower GI bleeding, with colonoscopy and CT often revealing colonic wall thickening, edema, and congestion. However, IMAV AVF is a rare etiological factor for IC. Despite its rarity, IMAV AVF induces IC through 2 primary mechanisms: a “steal phenomenon” reducing mucosal arterial perfusion, and venous hypertension causing congestive submucosal edema. Notably, IMAV AVF often precedes IC onset, many cases of IMAV AVF or AVMs exhibit mesenteric vasodilatation on CT prior to IC development. This time association, combined with the mechanistic evidence, supports that IMAVF plays a critical role in the pathogenesis of IC in patients where this fistula is present, even though it is not a common cause overall.

Angiography remains the gold standard diagnostic modality, enabling definitive assessment of whether IC is secondary to IMAV AVF.^[11]^ Early on, IMAV AVF appears angiographically as fistulae lesions in the blood-supplying vessels of the IMA. As the lesions progress, the marginal veins rapidly fill, dilate, and drain through the splenic, superior mesenteric, and portal veins, ultimately leading to occlusion of the inferior mesenteric vein.^[12]^ In addition, IMAV AVFs appear earlier than ICs, and many IMAV AVFs or AVMs present with mesenteric vasodilatation before ICs, which can be observed on CT. Therefore, early angiography can help to identify IC caused by IMAV AVF.^[13]^

The optimal management of IMAV AVFs remains a matter of debate and should be individualized, with treatment options generally including coil embolization or surgical resection.^[14]^ Coil embolization is effective, minimally invasive, and potentially safer by avoiding surgical intervention, with a relatively lower risk of hemorrhage.^[15]^ Nevertheless, it carries the risk of organ ischemia, particularly when multiple feeding arteries are embolized or when embolic material inadvertently enters the portal circulation. In contrast, surgical resection of the affected bowel together with the entire IMAV AVF carries risks of hemorrhage and incomplete exclusion of the fistula. According to available case reports, however, the success rate of surgical treatment has been reported as 100%, with no major complications observed. Therefore, surgical resection should be considered the preferred treatment option for patients with stable vital signs and in whom surgery is technically feasible.^[12,15]^ In the present case, our surgical team performed a thorough preoperative evaluation. CT revealed significant colonic edema, and colonoscopy demonstrated mucosal erosion with exudation. After multidisciplinary discussion, coil embolization was deemed to pose a high risk of postoperative ischemia; hence, surgical management was chosen. Intraoperatively, ischemic changes were identified in segments of the colon, further validating the appropriateness of surgical resection over embolization.

Furthermore, arteriography demonstrated that the patient had a large IMAV AVF diameter and a high flow rate (Fig. 5). We concluded that percutaneous endovascular embolization carries the risks of distal displacement of the embolic agent, colonic ischemia, and poor embolic effect due to multiple blood supply vessels. To quickly relieve the patient’s clinical symptoms, we chose surgical therapy.^[5,16]^

We recommend considering mesenteric AVFs in cases with clinical symptoms and signs of IC without a clear etiology. Prompt investigation and appropriate treatment strategies may reduce the risk of complications and postoperative morbidity and mortality from this condition.

## 4. Conclusion

This article presents a rare case of IMAV AVF with predominant clinical manifestations of watery and bleeding diarrhea without an apparent cause. Healthcare providers should consider this condition a potential diagnosis in patients exhibiting symptoms such as abdominal distension, abdominal pain, and bleeding diarrhea. A comprehensive approach, including prompt investigation and appropriate treatment strategies, is essential to reduce the risk of complications and improve patient outcomes. Future studies are needed to investigate the potential genetic and autoimmune associations of idiopathic IMAV AVF.

## Acknowledgments

The authors would like to thank each of the investigators who contributed to this article. We also sincerely appreciate the patient and his family for their cooperation in information acquisition, treatment, and follow-up.

## Author contributions

**Conceptualization**: Congcong Shi.

**Data curation**: Xuan Zheng.

**Supervision**: Yufeng Xing.

**Validation**: Shenfeng Wu.

**Visualization**: Congcong Shi, Shenfeng Wu.

**Writing – original draft**: Congcong Shi, Xuan Zheng, Lihong Chen.

**Writing – review & editing**: Shenfeng Wu, Ziyou Zhong, Yufeng Xing.
